# Sequencing, *de novo *annotation and analysis of the first *Anguilla anguilla *transcriptome: EeelBase opens new perspectives for the study of the critically endangered european eel

**DOI:** 10.1186/1471-2164-11-635

**Published:** 2010-11-16

**Authors:** Alessandro Coppe, Jose Martin Pujolar, Gregory E Maes, Peter F Larsen, Michael M Hansen, Louis Bernatchez, Lorenzo Zane, Stefania Bortoluzzi

**Affiliations:** 1 Biology Department, University of Padova, Via G. Colombo 3, I-35131 Padova, Italy; 2 Katholieke Universiteit Leuven, Laboratory of Animal Diversity & Systematics, B-3000 Leuven, Belgium; 3 Aarhus University, Department of Biological Sciences, Ny Munkegade 114, DK-8000 Aarhus C, Denmark; 4 University of Laval, IBIS, Quebec City, PQ G1V 0A6 Canada

## Abstract

**Background:**

Once highly abundant, the European eel (*Anguilla anguilla *L.; Anguillidae; Teleostei) is considered to be critically endangered and on the verge of extinction, as the stock has declined by 90-99% since the 1980s. Yet, the species is poorly characterized at molecular level with little sequence information available in public databases.

**Results:**

The first European eel transcriptome was obtained by 454 FLX Titanium sequencing of a normalized cDNA library, produced from a pool of 18 glass eels (juveniles) from the French Atlantic coast and two sites in the Mediterranean coast. Over 310,000 reads were assembled in a total of 19,631 transcribed contigs, with an average length of 531 nucleotides. Overall 36% of the contigs were annotated to known protein/nucleotide sequences and 35 putative miRNA identified.

**Conclusions:**

This study represents the first transcriptome analysis for a critically endangered species. EeelBase, a dedicated database of annotated transcriptome sequences of the European eel is freely available at http://compgen.bio.unipd.it/eeelbase. Considering the multiple factors potentially involved in the decline of the European eel, including anthropogenic factors such as pollution and human-introduced diseases, our results will provide a rich source of data to discover and identify new genes, characterize gene expression, as well as for identification of genetic markers scattered across the genome to be used in various applications.

## Background

The European eel (*Anguilla anguilla *L.; Anguillidae; Teleostei) is a catadromous fish species with a complex life cycle conditioned by marine (spawning, larval phase and maturation) and continental (feeding, growth) environments. Current available information indicates that the overall stock is at an historical minimum in most of the distribution area and continues to decline, while fishing mortality is still high both on juveniles (glass eels) and adults (yellow and silver eels) [1]. At present, recruitment is dramatically low, with a sharp and widespread reduction by 90-99% as compared to recruitment prior to 1980 [2]. Several hypotheses have been put forward concerning the causes of the eel stock decline, including anthropogenic factors affecting eels during their continental phase of the life-cycle (overfishing, migration barriers, pollution and human-introduced diseases; [3]) and climatic events affecting eels during the oceanic phase [4,5]. The European eel was included in 2007 in Appendix II of the Convention on International Trade of Endangered Species (CITES; http://www.cites.org) and was listed in 2008 as critically endangered in the IUCN Red List of Threatened Species http://www.iucnredlist.org. A management framework for the recovery of the European eel stock was established in 2007 by the Council of the European Union through a dedicated regulation (EU 1100/2007) for eel recovery and sustainable use of the stock requiring the preparation of national eel management plans from any Member States. Current demand for eels cannot be met by fisheries and relies on aquaculture instead, based on wild-caught juvenile eels as artificial reproduction of the species is not yet feasible [1].

From this perspective, an evaluation of European eel population genetic structure, genetic diversity, effective (spawning) population size, and possible evolutionary responses to anthropogenic environmental stress is crucial. Traditionally, these issues have been addressed by studying a limited number of markers due to the shortage of genomic sequence resources available for eels. No genome sequencing have been conducted for any anguillid species so far and all the species within the genus *Anguilla *are still poorly characterized at the molecular level. For the species *A. anguilla *only 232 proteins are available. Similarly, only 121 ESTs and 404 nucleotide sequences are known, the latter including the complete mitochondrial genome (NCBI databases 9/25/2010), encoding 13 peptides.

Next-generation sequencing techniques such as 454 pyrosequencing methodology allow for a massive characterization of expressed genes [6-10]. A complete characterization of Expressed Sequence Tags (ESTs) provides an overview of the transcriptome, i.e. those genes expressed in a given tissue at a given time [11]. Initially, pyrosequencing was restricted to model organisms [12-15] because of the short reads (100-200 bp) produced that make *de novo *genome assembly difficult without a reference genome. However, the more accurate base calling and deeper sequencing coverage of the 454 approach means that transcribed genes of non-model organisms can be characterized without a pre-existing sequence reference. Recently, 454 pyrosequencing has been successfully applied to large-scale EST sequencing in non-model organisms [16], including insects [17,18], plants [19-21] and corals [22]. In fish, characterized transcriptomes include the whitefish *Corenogus clupeaformis *[23], the eelpout *Zoarces viviparus *[24], the lake sturgeon *Acipenser fulvescens *[25] and the cichlid *Amphilophus sp. *[26]. Pyrosequencing of ESTs can be used to characterize gene expression, discover and identify new genes, providing a rich data resource for identification of novel Type I genetic markers (microsatellites and SNPs) for quantitative trait locus (QTL) and population genomic analyses. Up till now, only 196 ESTs are available for the Japanese eel [27] and 121 ESTs for the European eel [28]. More recently, EST sequencing of a normalized *A. anguilla *cDNA library produced by the European Marine Genomics Network of Excellence allowed to obtain 4,893 ESTs (795 contigs and 4,008 singletons), used for the identification of putatively selected microsatellites markers [29,30].

The non-coding portion of the transcriptome has been largely neglected in studies focusing on non-model organisms despite its emerging biological importance and the continuous discovery of novel classes of functional non-coding RNAs [31,32]. As an example, microRNAs (miRNAs) are small non-coding RNAs playing an important role in the regulation of gene expression in a wide range of biological processes, including cell differentiation, organogenesis and development, which have been found in a wide range of organisms, from plants to viruses and vertebrates (reviewed in [33]). The majority of fish miRNAs have been characterised in model species (360 for *Danio rerio*, 131 for *Fugu rubripes *and 132 for *Tetraodon nigroviridis*), with the exception of rainbow trout [34].

Here we present the European eel transcriptome, obtained by 454 FLX Titanium sequencing of over 300,000 ESTs from a normalized cDNA library, and assembly of reads in about 19,000 contigs, representing *bona fide *individual transcripts. An innovative aspect of our study is the identification of putative European eel miRNA sequences, by comparing reconstructed contig sequences with known Metazoan miRNAs hairpin precursor sequences. In summary, 36% of contigs were annotated by similarity to known protein or nucleotide sequences, plus 35 contigs matching miRNAs sequences known in different species were identified. A database (EeelBase) has been established that provides the first picture of the genomic transcriptional activity of this economically important but endangered species. The database will be updated in the future, if additional data becomes available.

## Results and discussion

### Contigs assembly and validation

A normalised cDNA library obtained from pooling equimolar amounts of total RNA from 18 glass eels was sequenced using the 454 Titanium platform. A single sequencing run from a single region produced 310,079 reads, with an average sequence length of 266 nucleotides (available at NCBI Short Read Archive SRA020995).

Using MIRA 3, sequence reads were assembled into contigs, representing European eel transcripts. A first run of assembly using 264,866 reads (85.4% of the total) produced a total of 28,459 sequences, consisting of 28,229 contigs and 230 singlets. The large majority of contigs (25,614 contigs or 91%) were assembled with high confidence, while the remaining 2,615 contigs were assembled despite the absence of a starting region covered by an "anchor" read with long overlap with many other reads. Plots in Additional Files 1 and 2 describe the distributions of length and average quality over all assembled sequences, and illustrate pair-wise relations between main sequence properties (see also Table 1A).

**Table 1:** Statistics describing the distributions of different properties of contig sequences.

264,866 reads		**Min**.	**1**^**st **^**Q**	Median	Mean	**3**^**rd **^**Q**	**Max**.
**(A) 28,459 contigs**	**Length**	40	279	409	**455.2**	565	2109
	
	**Number of reads**	1	2	4	**9.3**	10	436
	
	**Average coverage**	1.000	1.910	2.990	**4.574**	5.460	258.700
	
	**Average quality**	13	34	38	**41.66**	49	83

**(B) 19,631 contigs**	**Length**	200	355	452	**530.6**	652	2109
	
	**Average quality**	30	35	39	**44.8**	53	90
	
	**GC content**	21.86	36.72	41.06	**41.44**	45.93	67.94

Properties of contig sequences (A) obtained by the first run of assembly and (B) included in the final set representing the European eel transcriptome.

Due to the heuristic nature of the assembly process and previous reports of redundancy (different contig sequences belonging to the same transcript region) in sets of transcriptome contigs assembled with different methods [17], a second run of assembly was conducted using the previously obtained contigs and singlets as input. In this way, one quarter of contigs (7,510) were further assembled in 3,048 meta-contigs, with an increase of the average length from 455 (all contigs) to 783 nucleotides (meta-contigs). On average, meta-contigs included 2.47 contigs (from 2 to 11) and were covered by 43.2 reads.

A total of 23,997 sequences were obtained by merging all meta-contigs with the contigs and singlets from the first assembly not included in any meta-contig. The number of contigs remained stable over further reassembly (data not shown), which suggests that most redundancy had been eliminated. A further quality check was conducted for the final set of putative transcripts by selecting only those sequences at least 200 nucleotide long and with a minimum average sequence quality of 30 (corresponding to an average error rate of 1/1,000). A total of 19,631 transcripts were obtained (Table 1B), with an average length of 531 nucleotides and an average sequence quality of 45 (about 1 in 32,000 bp error rate). Transcripts included information derived from 248,011 original reads, with an average of 12.6 reads per transcript. The GC content in the transcripts ranged from 21.86% to 67.94%, with a mean value of 41.44% (median 41.06).

Figure in Additional File 3 shows the distribution of sequence length and average quality in the final set of contigs. All contigs were aligned with the set of original reads from which they were assembled, generating multiple alignments in ACE format. These were included in the database and might be useful for future identification of intra- and inter-specific sites of genetic variation (SNPs, microsatellites).

To our knowledge, no general criteria have been proposed as standard for quality evaluation of *de novo *transcriptome assembly. In this sense, three aspects can be regarded as substantial for assessing how well the sample of assembled contig sequences represents the actual transcriptome population: (1) gene coverage, (2) transcript sequence quality and (3) completeness.

(1) First, we compared the ratio between number of genes and transcripts in zebrafish *Danio rerio *and stickleback *Gasterosteus aculeatus*. A recent paper by Lu et al. [35] showed that rates of alternative splicing vary among teleost species, in terms of fraction of genes with alternative transcripts (17% in zebrafish; 32.4% in stickleback) and average number of splicing events per gene (1.74 in zebrafish and 1.65 in stickleback), resulting in a different ratio between transcripts and genes (1.13 in zebrafish, 46,571 transcripts/41,365 genes; 1.21 in stickleback, 28,071 transcripts/23,188 genes). Under the hypothesis that the number of genes and the transcripts/genes ratio in *A. anguilla *is similar to that estimated for stickleback, which is reasonable considering the highly duplicated nature of zebrafish genome [35], the total of 19,631 European eel transcripts would represent about 16,200 genes, with at best about 70% gene coverage.

The transcriptome gene coverage was estimated by comparison with the available sequence information for *A. anguilla. *All 13 mitochondrial protein-coding genes previously described in European eel were present in the assembled contigs. Moreover, regarding the 232 known *A. anguilla *protein sequences, 113 (75%) out of the 150 different proteins (after eliminating redundancy) were found in the transcriptome. These two estimations might be inaccurate because of the limited numbers of sequences used for comparison, mostly belonging to highly and/or constitutively expressed genes. By contrast, only 5 out of the 8 glutathione peroxidase genes known in zebrafish are found in the eel transcriptome. While 12 genes of two small families of Iroquois homeobox proteins are found in zebrafish, only 5 contigs/2 putative genes are represented in the eel transcripts. For ATPase genes, a large family in vertebrates with over 125 genes in zebrafish, only 15 contigs/9 putative genes were present in the eel transcriptome.

However, coverage values in the European eel transcriptome might not be comparable to zebrafish, the genome of which is characterized by high rates of gene duplications [35], which might correspond to a considerable increase in gene family size in comparison with other species. A moderate to low gene coverage can also be attributable to tissue/life stage-specific and/or weakly expressed gene transcripts, which might be applicable to our study in which the cDNA library was produced using a single life-stage (glass eels).

(2) Transcriptome sequence quality was evaluated by comparing the mitochondrial protein-coding genes found in the assembled contigs with the mitochondrion sequence in Genbank (NC_006531). A total of 18,554 nucleotide identities were observed out of 18,791 total nucleotide length of contig to genome BLAST matches, suggestive of good transcriptome sequence quality. The observed 1% sequence difference might be due to either intraspecific genetic variability and/or sequencing errors affecting assembled mtDNA sequences.

(3) Finally, in terms of sequence completeness, the estimation of the fraction of full-length sequences in the transcriptome was obtained. A sequence is considered full-length when it comprises the complete 5' and 3' sequences of the mRNA. In this study, we used a less stringent but broadly adopted definition, considering a sequence as full length when it contains at least the complete coding sequence (CDS). Using the software Full-Lengther, 54% of predicted transcripts were validated as full-length (10,169) or putative full-length (474). Approximately 25% (2,575) presented at least a significant BLAST match (min. E-value = 1E-3) with nucleotide (nr) or protein (UniProt) sequences. The remaining 75% (8,068) were not similar to any known sequence but their longest ORF fitted the Full-lengther criteria (exceeds 66 nucleotides and contains both ATG and stop codons, or the ATG is located at no more than 150 nucleotides from the 5' end of the transcript, without an in-frame end codon). Among the 8,988 transcripts considered as non full-length by the software, 2,348 (12% of the total) showed BLAST matches. Thus, at least 66% of all contigs (54% full-length plus 12% non-full-length but with BLAST hits) could be successfully annotated by similarity and/or contained a complete CDS.

### Functional annotation by similarity

*De novo *annotation of the European eel transcriptome, both for the coding and for the non-coding fraction of transcripts, was obtained by a multistep procedure, starting with similarity search against main protein and nucleotide sequence databases, as detailed below.

#### BLASTX against protein sequence databases

**Figure 1:**
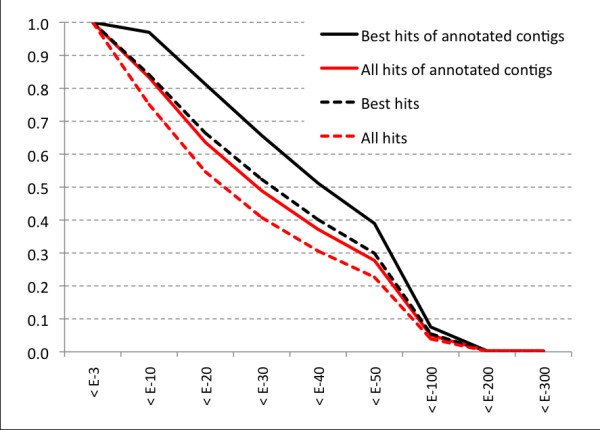
**BLAST E-values (Eel contigs *vs *nr protein database) distribution analysis**. Lines show the fraction of E-values lower than the threshold indicated in the x-axis. Four groups of HSP- associated E-values are considered, corresponding to the best hits only and to all the hits, both for the complete set of 5,530 contigs with protein BLAST hits and for the subset of 3,556 contigs with protein hits, which were subsequently annotated, by association to GO terms.

**Figure 2:**
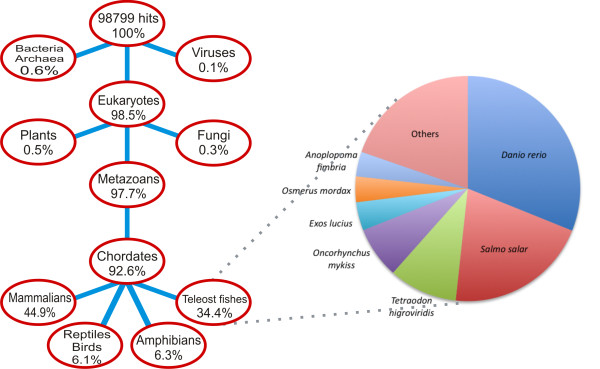
**Species distribution of European eel BLAST hits from nr sequence database**. A total of 5,530 contigs found significant similarity with 98,799 hit sequences. The relative abundance of hits in main taxonomic groups is represented as percentage of the total number of hits, in a simplified "tree of life" diagram (lines between nodes mean "belong to").

Transcript sequences were compared by BLASTX against nr database of peptide sequences, the most comprehensive and well annotated collection of proteins, thus identifying significant similarity with known proteins for 5,530 transcripts (28.2%). In total, 98,799 nr hits were identified, with an average of 18 hits per transcript. Figure 1 shows the European eel contigs *vs *nr protein database BLAST E-values analysis, in relation to the annotated status of contigs. Eukaryotes accounted for 98.5% of all BLAST hits, while teleost fish accounted for 34.4% of hits (Table 2; Figure 2). Among fish, zebrafish *Danio rerio *and salmon *Salmo salar *represented about 50% of all the hits, with 10,540 and 6,991 hits, respectively.

**Table 2:** E-values and Scores distribution of all the 155,749 alignments identified by BLAST between European eel transcripts and nr protein hits.

BLAST alignments	**Min**.	**1**^**st **^**Q**	Median	Mean	**3**^**rd **^**Q**	**Max**.
**E-value**	0	1.430E-47	8.743E-24	**1.928E-05**	9.604E-11	9.982E-04

**Score**	41	131	209	**289**	380	2155

Considering alignment coverage between query and subject sequences, aligned regions covered on average 45.1% of contigs length: 75% of contigs were aligned with subject sequences for more than 24.6% of their length, and 25% for more than 63.5% of their length. Aligned regions covered on average 35.1% of subject sequence (known proteins) length, whereas three quarters of aligned regions covered over 55.1% of subject sequence length. The majority of contig/transcript sequences (14,316) were not associated to nr BLAST hits. Comparison of sequence length, quality and GC content of the set of sequences with and without nr BLAST hits showed highly significant differences (Table 3): annotated sequences were longer and of higher quality than non-annotated sequences, and GC content of the two sets was on average about 8 percent points higher in annotated sequences.

**Table 3:** Comparison among contig sequences annotated by similarity search against nr database and contig sequences without any significant hit.

Average	Contigs with nr BLAST hits	Contigs without nr BLAST hits	t-test p-value
**Length**	666.8	479.2	<2.2E-16
	
**Average quality**	50.17	42.77	
	
**GC content**	46.97	39.34	

In parallel, BLASTX search with the SwissProt set of UniProt protein sequences identified significant similarity with known proteins for 4,023 transcripts (20.5%) with a total of 62,630 hits, with 16 hits per transcript on average. Only 29 contigs, not previously annotated by similarity using nr database, were included in the set of 4,023 contigs with SwissProt BLAST hits. Merging the results of the two BLAST searches, 5,559 transcripts (28.3%) resulted to be similar to at least one known protein sequence in UniProt or nr database, with adopted settings.

#### BLAST against nucleotide sequence databases

Transcript sequences were also compared by BLASTN against nt database of nucleotide sequences, identifying significant similarity for 5,495 transcripts (28%). In total, 70,530 nt hits were identified, with an average number of 13 hits per transcript. A group of 1,433 contigs without hits after BLASTX searches resulted to be similar to nt sequences.

In summary, a total of 6,963 contigs with at least one hit after all the BLAST searches were identified, which represent 36% of the transcriptome.

#### Functional annotation

**Figure 3:**
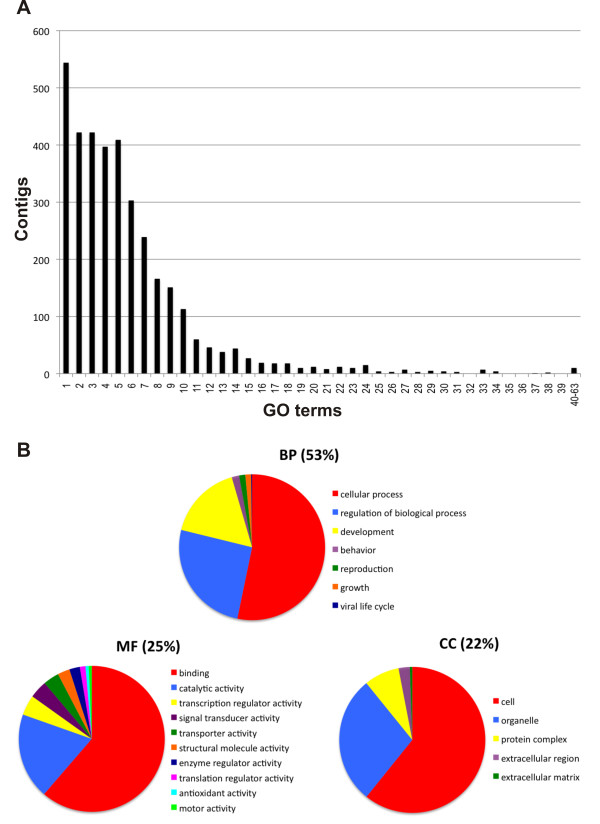
**Functional annotation**. Panel A reports the number of transcripts annotated with numbers of GO terms per transcripts. Panel B shows European eel contigs GO terms representation for biological process (BP), molecular function (MF) and cellular component (CC) ontologies, calculated after mapping by single count 122,193 GO terms to a total of 124 GO-Slim ancestor terms.

BLASTX with nr database was chosen as most informative and used as starting point for the functional annotation analysis conducted with the Blast2GO suite. Among contigs with nr BLASTX hits, 3,556 (64%) were associated to one or more 3,276 unique GO terms, for a total of 122,193 term occurrences. After merging GO annotations to eliminate redundancy, 18.1% of contigs resulted to be associated to GO terms. The number of GO terms per annotated contig is reported in Figure 3A. In order to give a broad overview of the ontology content, GO classes were grouped into GO-slim terms, which are cut-down versions of the GO ontologies containing a subset of the terms in the whole GO. Using the web tool CateGOrizer, GO classes were grouped into a total of 124 GO-Slim terms (Figure 3B, Additional File 4), which included biological process (53%), molecular function (25%) and cellular component (22%) ontologies. Among biological processes, cellular, regulatory and development processes represented 95% of the total, while other key processes like growth or reproduction were also present. Binding represented about 70% of the molecular function terms.

Sequence and annotation information included in EeelBase might be valuable for the study of European eel biology under changing environmental conditions. Different groups of European eel transcripts that putatively encode proteins critical for environmental stress response are found in the database. Several transcripts encoding proteins putatively involved in environmental adaptation were found in the database. A total of 11 different heat shock proteins were identified (Additional File 5), a class of functionally related proteins whose expression is increased when exposed to stress, many functioning as molecular chaperones with a critical role in protein binding and folding. Regarding oxidative stress response, 12 contigs encoding at least 5 different forms of glutathione peroxidase were identified, key enzymes involved in detoxification of hydrogen peroxide but also associated with SH3-domain binding, endopeptidase inhibition and anti-apoptotic activity through caspase regulation. Three contigs were annotated as encoding at least two superoxide dismutase proteins, a class of enzymes with a role in superoxide catalysis. Finally, 22 contigs represented MHC (Major Histocompatibility Complex) genes, which play an important role in the immune system. Direct keyword search by GO terms, implemented in the database, allows to efficiently retrieve the relevant information.

As a last step, the KEGG (Kyoto Encyclopedia of Genes and Genomes) pathway approach for higher order functional annotation was implemented using the tool DAVID. Using zebrafish as reference genome, a total of 2,076 zebrafish genes homologous to European eel transcripts were mapped to KEGG pathways. Three of them are significantly enriched: ribosome (37 genes, FDR 2.7E-9), oxidative phosphorylation (34 genes, FDR 2.4E-4) and proteasome (15 genes, FDR 2.1E-1) (Additional File 6).

### Comparison with zebrafish and three-spine stickleback genomes

European eel transcripts were aligned with the complete zebrafish genome using BLAT, in order to reconstruct the exon-intron structure of European eel genes, which might be useful for primer and probes design in future experimental studies. For each transcript, the pairwise alignment with the best-matching zebrafish genome region was retrieved and analysed. In total, BLAT detected similarities with a genomic region for 18,990 contigs, although a fraction of alignments included small regions and/or low percentages of sequence identity. Using alignments between transcript and genomic of at least 100 nucleotides and 70% sequence identity as criteria, 3,245 transcripts (17.1%) were related *bona fide *to the corresponding orthologous region in the zebrafish genome. Considering the ratio between matching region and transcript length, 77.8% of transcripts with significant genome match aligned with zebrafish for at least 50% of the sequence length, while 35.7% aligned with at least 75% of the sequence length. Similarly, eel transcripts were aligned with the available genome of the three-spine stickleback *Gasterosteus aculeatus*. Applying the same criteria used for zebrafish genome matches, only 1,062 European eel contigs, corresponding to 5.4% of the total, aligned with the stickleback genome. Of these, 4% aligned for at least 50% of the sequence length and 0.2% for at least 75% of the sequence length, whereas 671 (63%) aligned also with zebrafish genome.

According to phylogenetic data, Anguilliformes are a basal branch in the teleost evolution that diverged before the separation of other teleost lineages, including Cyproniformes (zebrafish) and Gasterosteiformes (stickleback). Thus, discrepancies in genome matches across species could be explained by differences in genome structure rather than by differential sequence divergence. The higher number of matches in zebrafish may be consequence of the highly duplicated nature of the zebrafish genome [35]. Indeed, genome size is three times larger in zebrafish (1.7 Gb) than in stickleback (0.6 Gb), according to [35]. Moreover, while the zebrafish genome is fully sequenced, the stickleback genome is under completion with only 0.45 Gb available so far (Ensembl assembly BROAD S1).

### Transcriptome redundancy

Transcriptome redundancy is expected in assembled contigs due to the heuristic nature of the assembly process and the settings used to avoid assembly of slightly different sequences. Different kinds of redundancy can be considered. Transcript-level redundancy is observed if different contigs belong to the same transcript. This may result from lack of conditions for merging read sequences for a same transcript in a unique contig, due to no sequence overlap or to sequencing errors. Gene-level redundancy is observed when different contig sequences belong to the same gene or transcriptional region. This may partially be explained by the pervasive existence of alternative transcripts. Recently, Lu et al. [35] suggested an inverse relation between genome size and alternative splicing frequency in teleost fish. While the lowest value of alternatively splicing was found in the highly duplicated zebrafish genome, the highest value was found in the compact genome of pufferfish. Assuming 30% alternative splicing that is intermediate among the values found in [35] and an approximate value of 1.7 events per gene, about 42% of transcripts are expected to belong to alternatively spliced genes in the European eel. Considering the fraction of annotated contigs, 5,314 contigs are associated to 3,202 unique descriptions, 2,281 of which are represented by a single contig. Thus 43% (2281/5314) of contigs might be transcripts of different genes, whereas the remaining fraction of contigs may be redundant at gene level (3.3 contigs per description, on average). Considering that different descriptions may correspond to different genes, the fraction of descriptions represented by at least two contigs (29%, 921/3202) is very close to the assumed 30% of alternatively spliced genes. On the other hand, the number of contigs per description exceeds the expected number of transcripts per alternatively spliced gene (3.3 and 1.7, respectively) with an excess only partially explained by the fact that some of the largest groups of contigs are associated to quite general descriptions (e.g. "novel protein", "histone" or "member ras oncogene family"). Thus, the description redundancy in European eel contig annotation may be largely, but not completely, explained by alternative splicing rates in fishes.

As an example, contig eu_c381 (2109 nucleotides long, average quality 66) is annotated as PSA2 (proteasome subunit alpha type-2), as it matches different proteins of the InterPro family IPR000426 (Proteasome, alpha-subunit, conserved site). The contig sequence corresponds to a putative full-length transcript with best match in translated nr to a putative ortholog of zebrafish (NP_001122146), a 234 aa proteasome subunit, whose CDS is completely included in the European eel transcriptome. The alignment of the same contig with the zebrafish genome highlights the existence of detectable sequence similarity also outside the CDS, since the contig aligns with a 4,930 nucleotides genomic region of chromosome 19, with a total of 1,448 nucleotides aligned with 87% sequence identity. Looking at the contig annotation, there are two additional (shorter) contigs in the transcriptome, which are annotated as PSA2: eu_c1147 and eu_c27369. These sequences match partially overlapping protein hits groups with eu_c381, and match the same zebrafish genome region. However, they include different combinations of sequence fragments and likely correspond to alternative transcripts. Thus, these three transcripts belonging to a putative PSA2 European eel gene are correctly included in separated contigs.

### Identification of putative microRNAs

MicroRNAs are small non-coding RNAs playing important roles in the regulation of gene expression in biological processes including cell differentiation, organogenesis and development [33]. In order to identify putative novel microRNAs belonging to evolutionary conserved families, the European eel transcriptome was compared with known metazoan microRNA hairpin sequences. A total of 54 significant local alignments between contigs and hairpin sequences were identified, involving 54 different hairpins. For each hairpin sequence, we also considered the absolute positions of the known major and/or minor mature sequences in the hairpin, given by the miRBase database, in order to discriminate between contig/hairpin matches involving a more or less extended region of a hairpin from those overlapping a mature miRNA sequence. Table 4 reports a total of 35 contigs matching both hairpins and mature miRNAs sequences known in different species.

**Table 4:** List of European eel contigs putatively including a microRNA.

European eel contig	miRNA		Species
eu2_c1597	sme-mir-2175	Major	*Schmidtea mediterranea*
eu2_c1626	ptr-mir-1282-2	Major	*Pan troglodytes*
eu2_c1815	mmu-mir-203	Major	*Mus musculus*
eu2_c1886	mmu-mir-2142	Major	*Mus musculus*
eu2_c1924	mmu-mir-682	Major	*Mus musculus*
eu_c793	mml-mir-297	Major	*Macaca mulatta*
eu_c2050	gga-mir-1814	Major	*Gallus gallus*
eu_c2515	sko-mir-252b	Major	*Saccoglossus kowalevskii*
eu_c3632	mmu-mir-466d	Minor	*Mus musculus*
eu_c4382	mmu-mir-1192	Major	*Mus musculus*
eu_c5018	sko-mir-252b	Major	*Saccoglossus kowalevskii*
eu_c5414	sme-mir-756	Minor	*Schmidtea mediterranea*
eu_c6355	mdo-let-7d	Major	*Monodelphis domestica*
eu_c6415	sme-mir-87d	Major	*Schmidtea mediterranea*
eu_c7274	mmu-mir-466i	Major	*Mus musculus*
eu_c7409	bta-mir-220e	Major	*Bos taurus*
eu_c7978	mmu-mir-1937a	Major	*Mus musculus*
eu_c8918	ptr-mir-297	Major	*Pan troglodytes*
eu_c9238	mml-mir-298	Major	*Macaca mulatta*
eu_c9980	mmu-mir-1192	Major	*Mus musculus*
eu_c10632	mmu-mir-2142	Major	*Mus musculus*
eu_c11528	dgr-mir-308	Major	*Drosophila grimshawi*
eu_c11553	rno-mir-297	Major	*Rattus norvegicus*
eu_c12746	hsa-mir-522	Major	*Homo sapiens*
eu_c14091	hsa-mir-224	Minor	*Homo sapiens*
eu_c14597	mmu-mir-1903	Major	*Mus musculus*
eu_c14993	mmu-mir-2142	Major	*Mus musculus*
eu_c16240	bta-mir-2444	Major	*Bos taurus*
eu_c17072	mmu-mir-669i	Major	*Mus musculus*
eu_c17332	rno-mir-124-2	Major	*Rattus norvegicus*
eu_c17397	hsa-mir-297	Major	*Homo sapiens*
eu_c17444	dan-mir-92a	Major	*Drosophila ananassae*
eu_c18441	dre-mir-107b	Major	*Danio rerio*
eu_c19859	mmu-mir-674	Major	*Mus musculus*
eu_c20136	dan-mir-289	Major	*Drosophila ananassae*

Contigs were selected when part of their sequence is highly similar to a known Metazoan hairpin microRNA precursor. For each hairpin sequence, we also considered if the regions aligned with the hairpin sequence overlap the major or the minor mature sequence position.

### EeelBase: the European eel transcriptome database

**Figure 4:**
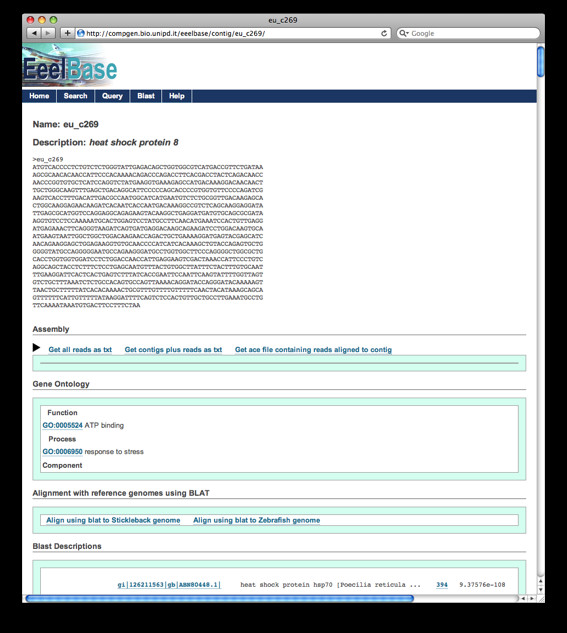
**EeelBase screenshots**. Example of the "gene-like" entry in the European eel transcriptome database (EeelBase). For each contig, different categories of information are given together with links to additional web pages (e.g. UCSC graphic display of contig alignments with zebrafish genome).

A database, freely available online at http://compgen.bio.unipd.it/eeelbase/, has been implemented using MySQL and Django web framework. The database is filled with different layers of information regarding the European eel transcriptome sequences and analysis results. For each contig, a gene-like entry (Figure 4) reports different data and bioinformatic analyses results, according to the schema detailed below:

• Contig information. For each contig (identified by EeelBase ID and preliminary description), the FASTA sequence is provided along with an informative contig description using Blast2GO or the best hit when the Blast2GO description was unavailable.

• Assembly. The list of reads belonging to the contig is given, together with two FASTA files including all read sequences, contig with read sequences, and multiple alignment of the contig with reads.

• Gene Ontology. GO terms associated to transcripts using the Blast2GO analysis on BLASTX vs nr database results are given for the three ontologies, linked to the GO database. BLAST results, both for nucleotide and protein database searches, are shown in a dedicated section in the classic BLAST output format, hyperlinked to external databases, and including the list of alignment descriptions and details about the pairwise alignments of the transcript with BLAST hits.

• Reference fish genomes alignment. Zebrafish and three-spine stickleback genome matches in the UCSC Genome Browser are provided to the user, by means of links allowing *on the fly *BLAT search against the last release of zebrafish and three-spine stickleback genomes, thus facilitating the identification and visualisation of one or more genomic regions putatively homologous to the considered transcript.

• BLAST results. Both for nucleotide and protein database, results of similarity searches are shown in a dedicated section in the classic BLAST output format, including the list of alignments descriptions and details about the pairwise alignments with hits, each hyperlinked to external databases entries.

• Putative miRNAs. For those eel transcripts including a putative miRNA sequence, a dedicated field is included in the entry, detailing its identity, linked to the corresponding miRBase database entry.

Summary of EeelBase information content (Table 5) is reported in the home page, which will be regularly updated with the subsequent database releases.

**Table 5:** Summary of the annotation data included in the first release of EeelBase.

Contigs	Number	Percentage
**Total**	19631	100

**Putative Full-length**	10643	54.2

**With BLAST hits**	6963	35.5
***nr hits***	*5530*	*28.2*
***SwissProt hits***	*4023*	*20.5*
***nt hits***	*5495*	*28.0*

**With GO terms**	3556	18.1

**Predicted microRNAs**	35	0.2

**Novel/hypothetical**	12640	64.4

The database is searchable by keywords and by BLAST, using nucleotide or protein sequences. Indeed, it implements a query system for massive data retrieval. For a given group of contigs, selected by GO terms ID or by keywords search on contigs and BLAST hits descriptions, a customizable .tsv file can be retrieved with data regarding contig ID, description and sequence as well as associated GO IDs and terms. FASTA files and ACE files with reads/contigs alignments can be downloaded from the main page.

## Conclusions

Next generation sequencing has opened the door to genomic analysis of non-model organisms. The growing number of species for which significant genetic resources are available is sparking a new era of study in which fundamental genetic questions underlying phenotypic evolution, adaptation and speciation can be addressed with rigor. The European eel transcriptome, the first obtained by high throughput 454 sequencing for a critically endangered species, has been produced, annotated and made freely available through a dedicated and searchable database. With over 19,000 contigs, 36% of which annotated by similarity to known protein or nucleotide sequences and about 18.5% aligned to the zebrafish or three-spine stickleback genomes, and 35 contigs matching miRNAs sequences known in different metazoan species, this new resource represents a significant advance in anguillid genomics. Considering the critically endangered status of the European eel and the multiple factors potentially involved in eel decline, including anthropogenic factors such as pollution and human-introduced diseases, our results provide a rich source of data to discover and identify new genes, characterize gene expression and for the identification of microsatellites and single nucleotide polymorphisms (SNPs). Transcriptome sequencing is frequently used to provide greater insight into many basic biological questions. Applications include the understanding of adaptation, effects of and possible adaptive evolutionary responses to pollutants and other types of environmental stress, improvement of aquaculture, and the discovery of novel genes coding for important life-history traits.

Current demand for eels cannot be met by fisheries and relies on aquaculture instead [1]. The most promising application of genomics in the European eel is aquaculture, which currently satisfies the big market demand for eels that fisheries are no longer meeting. Using a proteomic approach, key genes for growth and survival in aquaculture stocks can be identified. In this sense, our annotation revealed several genes with a potential role in growth including growth hormone (GH), insulin-like growth factor (IGF) and transforming growth factor (TGF), primary candidates for genetic factors affecting growth. Our annotation also showed proteins related to stress, which could also be important in aquaculture as the reduction of stress might lead to a higher growth and reproductive output.

## Methods

### Biological samples

Recruiting glass eel (juvenile) samples were collected in early 2007 at three separate geographic locations across Europe, one in the Atlantic Ocean: (1) the estuary of the river Vilaine (47°29'N; 2°28'W) in Brittany/North-West France; and two in the Tyrrhenian Sea/Mediterranean Sea: (2) the estuary of the river Tiber (41°46'N; 12°14'E); and (3) the estuary of the river Sele (40°48'N; 14°93'E). Individuals were sacrificed immediately after collection from the field following internationally recognized guidelines. Samples were stored in RNALater (Ambion) at -20°C prior extraction. No analyses or experiments were conducted with live animals.

### RNA extraction and cDNA library construction

Total RNA was extracted from a total of 18 individuals (6 per sampling location) using the RNeasy mini-column kit (QIAGEN). To avoid overrepresentation of muscle specific genes only the cephalic region (approximately 30 mg) was used. After checking the integrity and size distribution of total RNA, RNA samples were pooled and stored in pure ethanol for shipment to the Max Planck Institute (Berlin, Germany). One single cDNA library was constructed using equal amounts of RNA and normalized for later sequencing. The SMART (Switching Mechanism At 5' end of RNA Template) kit from BD Biosciences Clontech was used to construct the cDNA libraries, which were later normalised using the duplex-specific nuclease (DSN) method [36].

### Sequencing

Approximately 15 μg of normalized cDNA were used for sequencing library construction at the Max Planck Institute, following described procedures [6]. Sequencing was performed using GS FLX Titanium series reagents and utilizing one single region on a Genome Sequencer FLX instrument. Bases were called with 454 software by processing the pyroluminescence intensity for each bead-containing well in each nucleotide incorporation and reads were trimmed to remove adapter sequences.

### Assembly

Sequence reads were assembled into contigs by using the MIRA 3 assembler [37], which uses iterative multipass strategies centered on high-confidence regions within sequences and uses low-confidence regions when needed, with special functions to assemble high numbers of highly similar sequences without prior masking. Two runs of assembly were conducted by MIRA 3 in "EST" and "accurate" usage mode, respectively. Settings adopted for the first run (*de novo *assembly) of ESTs were those defined by the 454 sequencing technology. [mira -project = eu1 -job = denovo,est,accurate,454 -notraceinfo]. The second run was conducted on previously obtained contigs, which were used as input for MIRA 3 as Sanger sequences. [mira -project = eu2 -job = denovo,est,accurate]. The complete set of available reads was realigned to contigs using the mapping assembly method provided by the Roche GS Reference Mapper, specifically designed for consensus alignment of reads against a given reference sequence.

### Putative full-length transcripts identification

The software Full-Lengther [38] was used to obtain a first validation of the assembly, integrating the results of BLAST against UniProt and nr with those of ORF prediction, in order to calculate the fraction of transcripts, i.e. assembled contigs, which can be considered *bona fide *full-length.

### BLAST against sequences databases and functional annotation

*De novo *functional annotation of the European eel transcriptome was obtained by similarity using BLAST, Blast2GO and custom made scripts. Batch BLAST similarity searches for the entire transcriptome were locally conducted against (1) nr peptide database (release of October 4 2009, including all non-redundant GenBank CDS translations + PDB + SwissProt + PIR+PRF); (2) the SwissProt part of the UniProt database; (3) nt database. BLASTX and BLASTN searches were carried out using default parameters. Alignments with an E-value < 1E-3 were considered significant.

The Blast2GO suite [39] was used for functional annotation of transcripts applying the function for the mapping of GO terms to transcripts with BLAST hits obtained from BLAST searches against nr. Only ontologies obtained from hits with E-value < 1E-6, annotation cut-off > 55, and a GO weight > 5 were used for annotation. The web tool CateGOrizer [40] was used for grouping and counting GO classes using the GO-Slim method [41]. Additionally, the KEGG (Kyoto Encyclopedia of Genes and Genomes, [42]) database, a knowledge base for systematic analysis of gene functions linking genomic and higher order functional information, was implemented for further functional annotation using the tool DAVID [43].

### BLAT against zebrafish and three-spine stickleback genomes

European eel transcripts were aligned with the complete genomes of zebrafish *Danio renio *and three-spine stickleback *Gasterosteus aculeatus *using the BLAT search tool in the UCSC (University of California Santa Cruz) Genome Browser. For all transcripts, the pairwise alignment with the best matching genome region was retrieved and recorded for statistical analysis.

### MicroRNA discovery

After transcription, primary miRNAs (pri-miRNAs) are cleaved by the microprocessor complex to generate precursors sequences with hairpin structure. These pre-miRNAs are exported from the nucleus and subsequently cleaved by Dicer to generate a miRNA-miRNA* duplex with an average length of 21 nucleotides. Finally, one of the two mature miRNAs is integrated into the miRISC (microRNA induce silencing) complex. By imperfect base pairing with the 3' untranslated region (3'-UTR) of their target mRNAs, mature miRNAs can cause target silencing mainly by translation inhibition or mRNA cleavage.

The complete set of microRNA hairpin sequences was downloaded from miRBase database release 14 [44], a searchable database of published miRNA sequences. The 10,867 sequences belonging to Metazoan species were compared to European eel contigs by BLAST similarity search, using the same thresholds and settings adopted before.

## Authors' contributions

AC carried out all the bioinformatic analyses, designed and implemented the database. JMP tested the database, contributed to the statistical analysis and interpretation of data and wrote the paper with SB. GEM, PFL, MMH and LB made substantial contributions to the conception of the study, acquisition of data, and were involved in revising the manuscript critically; LZ conceived the study, performed initial molecular work, acquired data, has been involved in all the phases of the project and reviewed the manuscript. SB participated to the bioinformatic analyses, performed the statistical analyses, interpreted results and wrote the paper with JMP. All the authors read and approved the final manuscript.

## Supplementary Material

Additional file 1 **Additional Figure**. Distribution of average quality (A), length (B) and number of reads (C) in the set of 28,229 contigs obtained by the first run of reads assembly. Click here for file

Additional file 2 **Additional Figure**. Pair-wise relationships between main properties (sequence length, number of reads per contig, average sequence quality, and average sequence coverage) characterizing the set of 28,229 contigs obtained by the first run of reads assembly. Click here for file

Additional file 3 **Additional Figure**. Distribution of sequence length (A) and relationship between length and average quality (B) in the set of 19,631 contigs of the European eel transcriptome. Click here for file

Additional file 4 **Additional Table**. Mapping of the 122,193 GO terms associated to the European eel contigs to a total of 124 GO-Slim ancestor terms by single count. Click here for file

Additional file 5 **Additional Table**. Genes encoding heat shock proteins represented in EeelBase. The table summarizes a total of 92 GO terms associated to 26 contigs belonging to 11 different heat shock protein genes. The 55 non-redundant terms are also reported in a word cloud form, with character size proportional to the number of occurrences of the functional term. Click here for file

Additional file 6 **Additional Figures**. Mapping of zebrafish genes homologous to European eel transcripts to three KEGG pathways: ribosome (37 genes), oxidative phosphorylation (34 genes) and proteasome (15 genes). Green boxes represent KEGG nodes specific to the considered organism; Red stars indicate enriched nodes, which may represent one or more genes. Click here for file
