# Supplementary figures and images for: Sequencing, de novo annotation and analysis of the first Anguilla anguilla transcriptome: EeelBase opens new perspectives for the study of the critically endangered european eel
Source: BMC Genomics. 2010 Nov 16;11:635. doi: 10.1186/1471-2164-11-635 (PMC3012609; doi:10.1186/1471-2164-11-635)

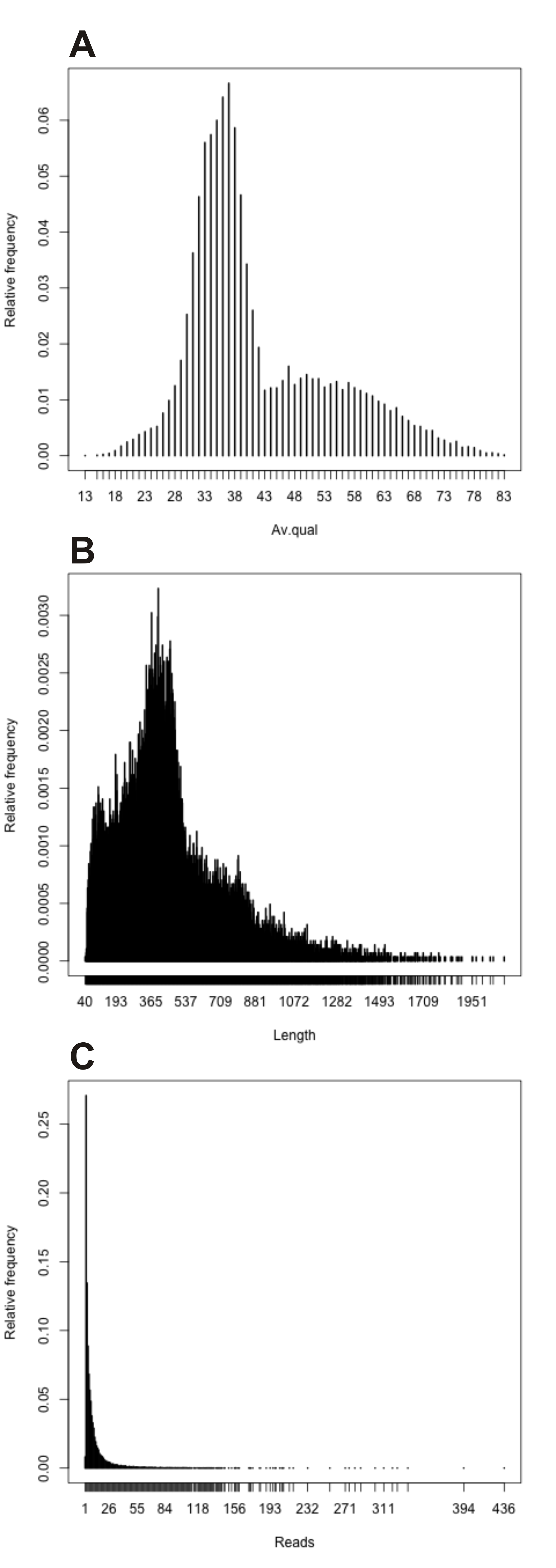

Supplement: Additional file 1 — Additional Figure. Distribution of average quality (A), length (B) and number of reads (C) in the set of 28,229 contigs obtained by the first run of reads assembly. [file 1471-2164-11-635-S1.PNG]

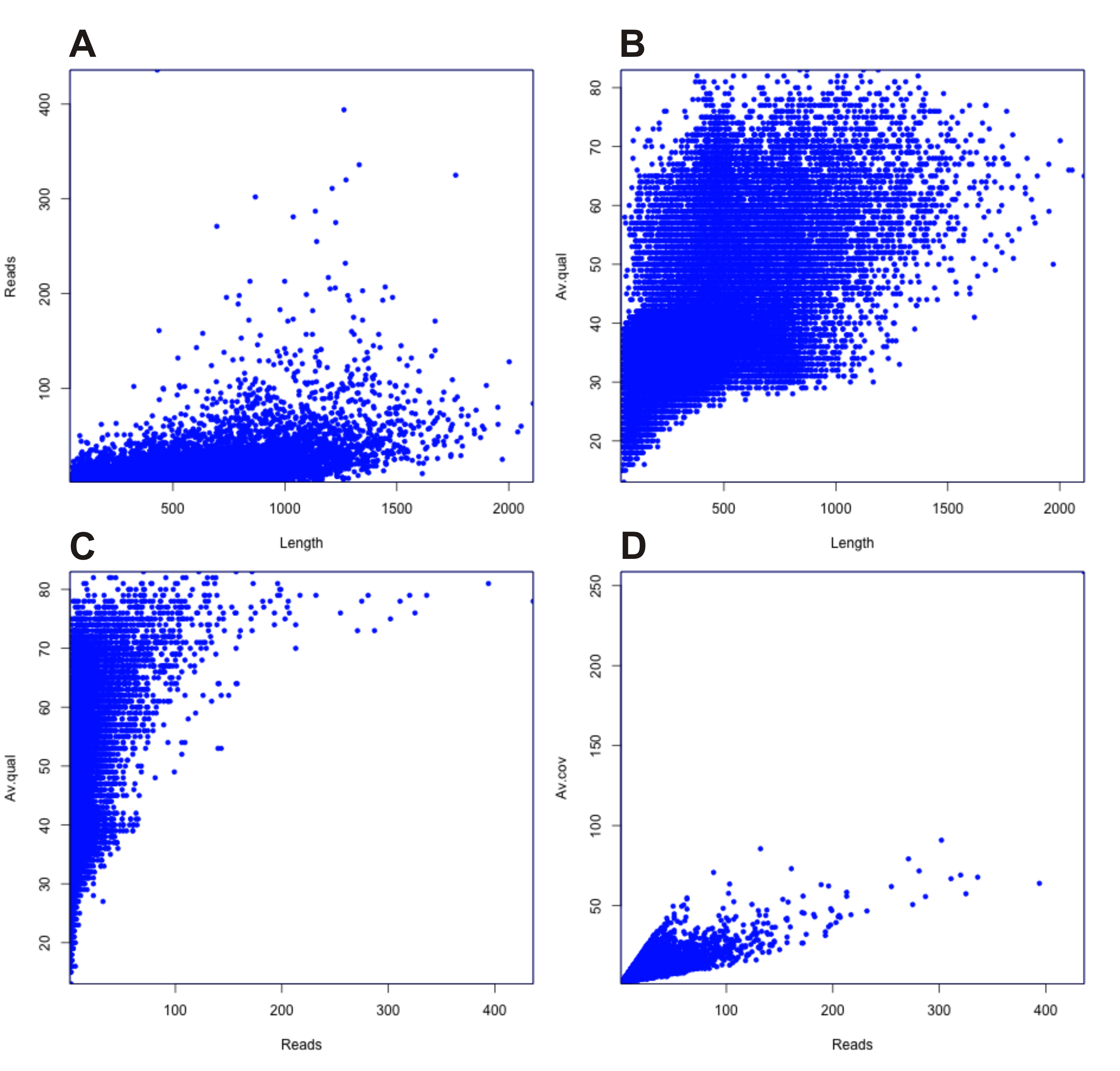

Supplement: Additional file 2 — Additional Figure. Pair-wise relationships between main properties (sequence length, number of reads per contig, average sequence quality, and average sequence coverage) characterizing the set of 28,229 contigs obtained by the first run of reads assembly. [file 1471-2164-11-635-S2.PNG]

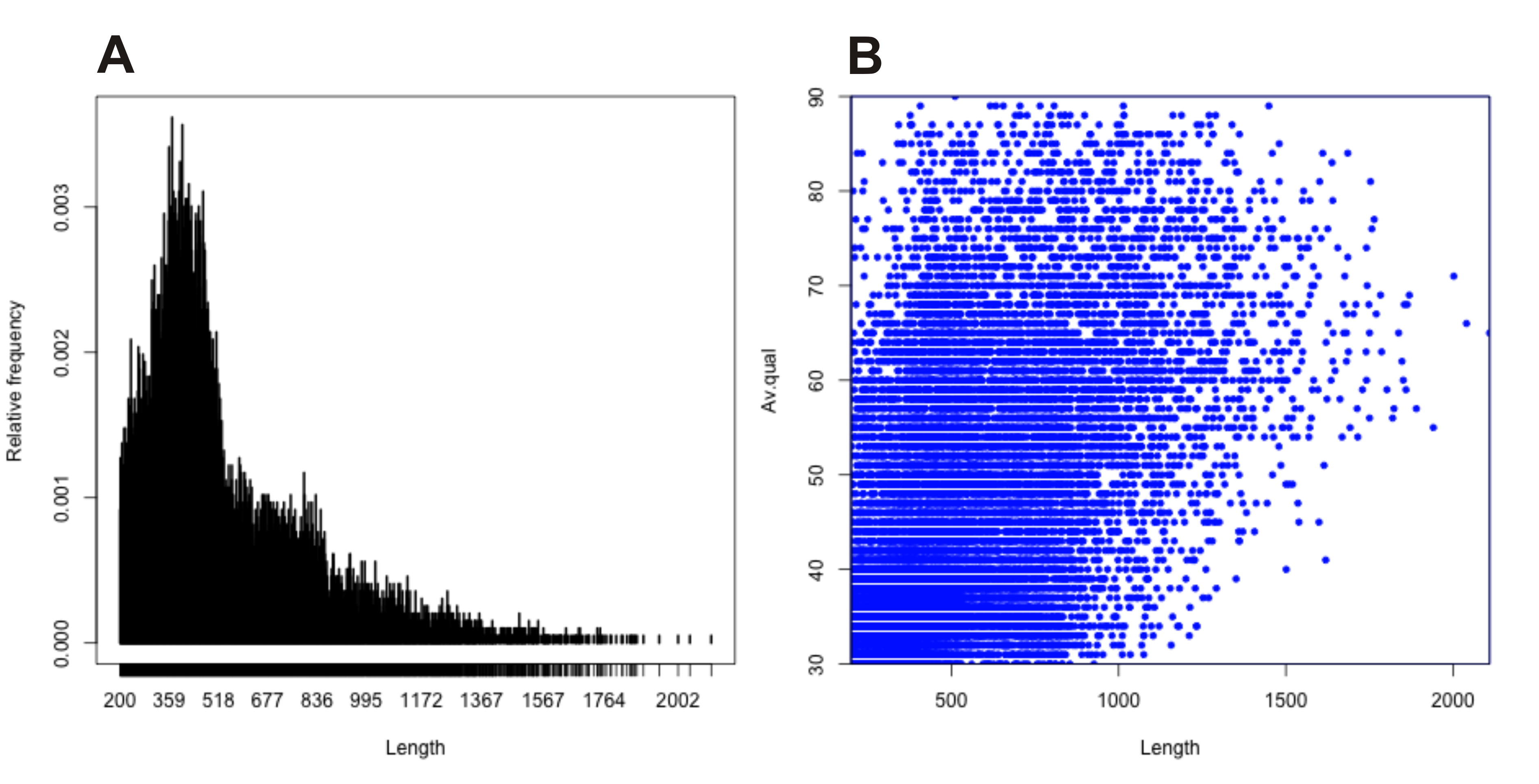

Supplement: Additional file 3 — Additional Figure. Distribution of sequence length (A) and relationship between length and average quality (B) in the set of 19,631 contigs of the European eel transcriptome. [file 1471-2164-11-635-S3.PNG]
