# Supplementary material for: Sequencing, de novo annotation and analysis of the first Anguilla anguilla transcriptome: EeelBase opens new perspectives for the study of the critically endangered european eel
Source: BMC Genomics. 2010 Nov 16;11:635. doi: 10.1186/1471-2164-11-635 (PMC3012609; doi:10.1186/1471-2164-11-635)
Supplement: Additional file 4 — Additional Table. Mapping of the 122,193 GO terms associated to the European eel contigs to a total of 124 GO-Slim ancestor terms by single count. [file 1471-2164-11-635-S4.DOC]

**Additional Table.** Mapping of the 122,193 GO terms associated to the European eel contigs to a total of 124 GO-Slim ancestor terms by single count.

| **GO Class ID** | **Definitions** | **#** | **%** |
| --- | --- | --- | --- |
| GO:0005623 | cell | 25708 | 10.08 |
| GO:0005488 | binding | 19759 | 7.75 |
| GO:0005622 | intracellular | 18480 | 7.25 |
| GO:0008152 | metabolism | 15334 | 6.01 |
| GO:0007275 | development | 12272 | 4.81 |
| GO:0005737 | cytoplasm | 11258 | 4.41 |
| GO:0005515 | protein binding | 9726 | 3.81 |
| GO:0016043 | cell organization and biogenesis | 6186 | 2.43 |
| GO:0003824 | catalytic activity | 6145 | 2.41 |
| GO:0007154 | cell communication | 6118 | 2.40 |
| GO:0009653 | morphogenesis | 5696 | 2.23 |
| GO:0009058 | biosynthesis | 5400 | 2.12 |
| GO:0006139 | nucleobase, nucleoside, nucleotide and nucleic acid metabolism | 5372 | 2.11 |
| GO:0019538 | protein metabolism | 5158 | 2.02 |
| GO:0030154 | cell differentiation | 5067 | 1.99 |
| GO:0006810 | transport | 4385 | 1.72 |
| GO:0007165 | signal transduction | 4266 | 1.67 |
| GO:0005634 | nucleus | 3405 | 1.34 |
| GO:0003676 | nucleic acid binding | 2937 | 1.15 |
| GO:0006996 | organelle organization and biogenesis | 2921 | 1.15 |
| GO:0016787 | hydrolase activity | 2856 | 1.12 |
| GO:0006950 | response to stress | 2848 | 1.12 |
| GO:0005829 | cytosol | 2809 | 1.10 |
| GO:0006350 | transcription | 2681 | 1.05 |
| GO:0016265 | death | 2509 | 0.98 |
| GO:0008219 | cell death | 2509 | 0.98 |
| GO:0005856 | cytoskeleton | 2435 | 0.95 |
| GO:0009790 | embryonic development | 2377 | 0.93 |
| GO:0007049 | cell cycle | 2080 | 0.82 |
| GO:0006464 | protein modification | 2005 | 0.79 |
| GO:0009056 | catabolism | 1896 | 0.74 |
| GO:0009605 | response to external stimulus | 1777 | 0.70 |
| GO:0005886 | plasma membrane | 1739 | 0.68 |
| GO:0007267 | cell-cell signaling | 1719 | 0.67 |
| GO:0003677 | DNA binding | 1686 | 0.66 |
| GO:0005739 | mitochondrion | 1545 | 0.61 |
| GO:0000166 | nucleotide binding | 1515 | 0.59 |
| GO:0007010 | cytoskeleton organization and biogenesis | 1496 | 0.59 |
| GO:0008283 | cell proliferation | 1480 | 0.58 |
| GO:0030528 | transcription regulator activity | 1439 | 0.56 |
| GO:0016740 | transferase activity | 1391 | 0.55 |
| GO:0004871 | signal transducer activity | 1271 | 0.50 |
| GO:0008092 | cytoskeletal protein binding | 1251 | 0.49 |
| GO:0009628 | response to abiotic stimulus | 1210 | 0.47 |
| GO:0007610 | behavior | 1205 | 0.47 |
| GO:0019725 | cell homeostasis | 1176 | 0.46 |
| GO:0005215 | transporter activity | 1133 | 0.44 |
| GO:0006259 | DNA metabolism | 1124 | 0.44 |
| GO:0005576 | extracellular region | 1054 | 0.41 |
| GO:0006629 | lipid metabolism | 1023 | 0.40 |
| GO:0000003 | reproduction | 1014 | 0.40 |
| GO:0009719 | response to endogenous stimulus | 996 | 0.39 |
| GO:0040007 | growth | 949 | 0.37 |
| GO:0015031 | protein transport | 896 | 0.35 |
| GO:0005198 | structural molecule activity | 870 | 0.34 |
| GO:0005654 | nucleoplasm | 844 | 0.33 |
| GO:0005509 | calcium ion binding | 828 | 0.32 |
| GO:0006412 | protein biosynthesis | 827 | 0.32 |
| GO:0005730 | nucleolus | 797 | 0.31 |
| GO:0005783 | endoplasmic reticulum | 777 | 0.30 |
| GO:0005794 | Golgi apparatus | 772 | 0.30 |
| GO:0030234 | enzyme regulator activity | 751 | 0.29 |
| GO:0006091 | generation of precursor metabolites and energy | 750 | 0.29 |
| GO:0016023 | cytoplasmic membrane-bound vesicle | 744 | 0.29 |
| GO:0005975 | carbohydrate metabolism | 721 | 0.28 |
| GO:0016301 | kinase activity | 693 | 0.27 |
| GO:0006811 | ion transport | 692 | 0.27 |
| GO:0005102 | receptor binding | 683 | 0.27 |
| GO:0003723 | RNA binding | 668 | 0.26 |
| GO:0005840 | ribosome | 625 | 0.25 |
| GO:0005694 | chromosome | 617 | 0.24 |
| GO:0004872 | receptor activity | 608 | 0.24 |
| GO:0009607 | response to biotic stimulus | 582 | 0.23 |
| GO:0008233 | peptidase activity | 537 | 0.21 |
| GO:0006519 | amino acid and derivative metabolism | 531 | 0.21 |
| GO:0003700 | transcription factor activity | 487 | 0.19 |
| GO:0003779 | actin binding | 469 | 0.18 |
| GO:0004672 | protein kinase activity | 455 | 0.18 |
| GO:0008289 | lipid binding | 440 | 0.17 |
| GO:0005615 | extracellular space | 428 | 0.17 |
| GO:0045182 | translation regulator activity | 403 | 0.16 |
| GO:0008135 | translation factor activity, nucleic acid binding | 384 | 0.15 |
| GO:0005773 | vacuole | 352 | 0.14 |
| GO:0007005 | mitochondrion organization and biogenesis | 331 | 0.13 |
| GO:0005764 | lysosome | 307 | 0.12 |
| GO:0016049 | cell growth | 304 | 0.12 |
| GO:0004721 | phosphoprotein phosphatase activity | 263 | 0.10 |
| GO:0005815 | microtubule organizing center | 261 | 0.10 |
| GO:0005768 | endosome | 243 | 0.10 |
| GO:0005929 | cilium | 233 | 0.09 |
| GO:0016209 | antioxidant activity | 231 | 0.09 |
| GO:0003774 | motor activity | 214 | 0.08 |
| GO:0005578 | extracellular matrix (sensu Metazoa) | 188 | 0.07 |
| GO:0004518 | nuclease activity | 180 | 0.07 |
| GO:0005216 | ion channel activity | 168 | 0.07 |
| GO:0005777 | peroxisome | 166 | 0.07 |
| GO:0005635 | nuclear membrane | 159 | 0.06 |
| GO:0030246 | carbohydrate binding | 153 | 0.06 |
| GO:0016032 | viral life cycle | 150 | 0.06 |
| GO:0000228 | nuclear chromosome | 123 | 0.05 |
| GO:0019748 | secondary metabolism | 116 | 0.05 |
| GO:0040029 | regulation of gene expression, epigenetic | 77 | 0.03 |
| GO:0008037 | cell recognition | 66 | 0.03 |
| GO:0003682 | chromatin binding | 23 | 0.01 |
| GO:0005326 | neurotransmitter transporter activity | 13 | 0.01 |
| GO:0007028 | cytoplasm organization and biogenesis | 9 | 0.00 |
| GO:0005811 | lipid particle | 7 | 0.00 |
| GO:0019825 | oxygen binding | 4 | 0.00 |
| GO:0009536 | plastid | 4 | 0.00 |
| GO:0030312 | external encapsulating structure | 1 | 0.00 |
| GO:0030313 | cell envelope | 1 | 0.00 |
| **Total** | | **255017** | **100.00%** |
