# Supplementary material for: Sequencing, de novo annotation and analysis of the first Anguilla anguilla transcriptome: EeelBase opens new perspectives for the study of the critically endangered european eel
Source: BMC Genomics. 2010 Nov 16;11:635. doi: 10.1186/1471-2164-11-635 (PMC3012609; doi:10.1186/1471-2164-11-635)
Supplement: Additional file 6 — Additional Figures. Mapping of zebrafish genes homologous to European eel transcripts to three KEGG pathways: ribosome (37 genes), oxidative phosphorylation (34 genes) and proteasome (15 genes). Green boxes represent KEGG nodes specific to the considered organism; Red stars indicate enriched nodes, which may represent one or more genes. [file 1471-2164-11-635-S6.PDF]

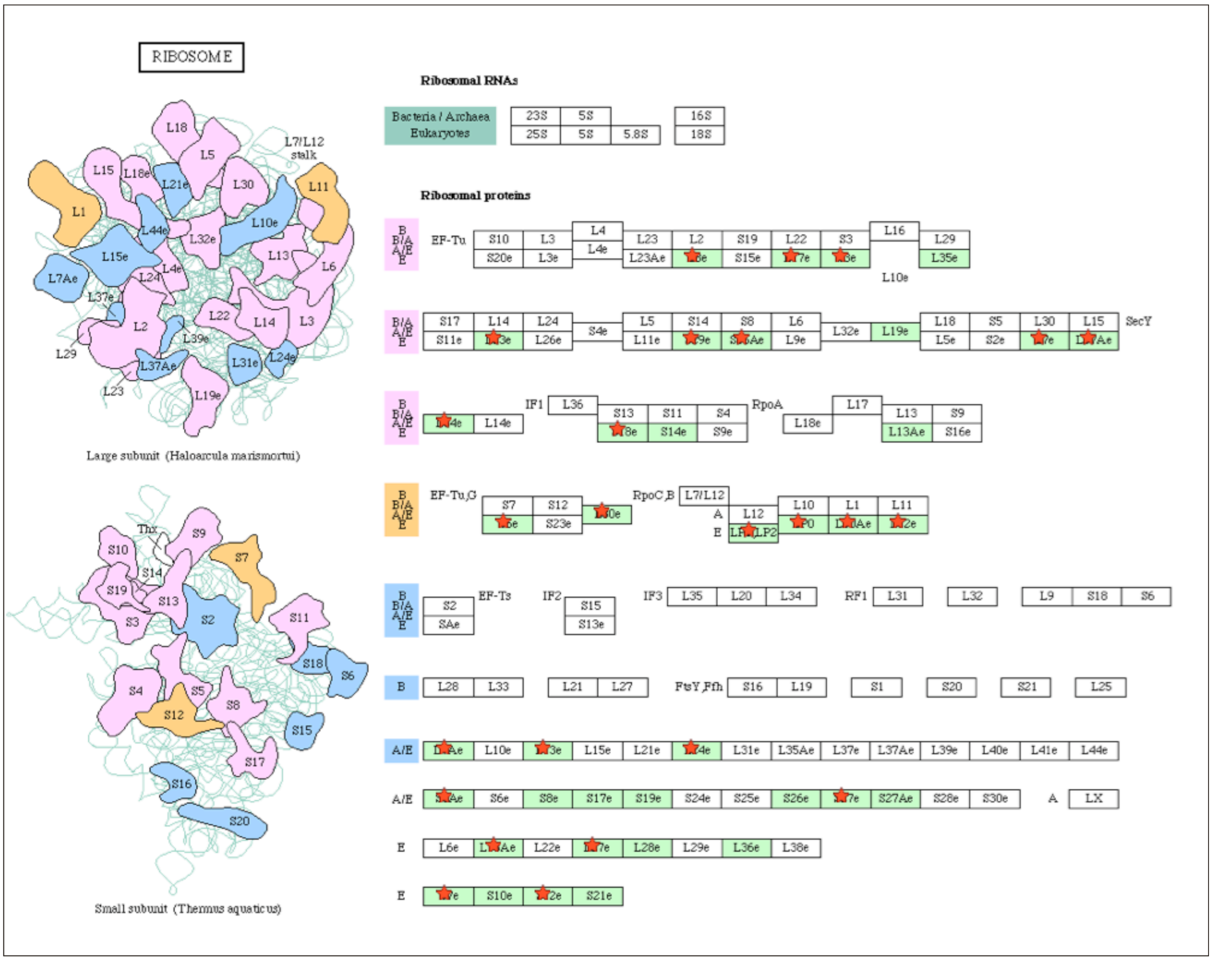

RIBOSOME
Ribosomal RNAs
Bacteria / Archaea
Eukaryotes
23S 5S 16S
25S 5S 5.8S 18S
Ribosomal proteins
EF-Tu
S10 L3 L4 L23 L2 S19 L22 S3 L16 L29
S20e L3e L4e L23Ae S15e L35e
L10e
S17 L14 L24 S4e L5 S14 S8 L6 L32e L19e L18 S5 L30 L15 SecY
S11e L26e L11e L9e L5e S2e
IF1 L36 RpoA L17
L14e S13 S11 S4 L18e L13 S9
S14e S9e L13Ae S16e
EF-Tu,G RpoC,B L7/L12
S7 S12 L12 L10 L1 L11
S23e A E
EF-Ts IF2 IF3 RF1
S2 S15 L35 L20 L34 L31 L32 L9 S18 S6
SAe S13e
B
L28 L33 L21 L27 FtsY,Ffh S16 L19 S1 S20 S21 L25
A/E
L10e L15e L21e L31e L35Ae L37e L37Ae L39e L40e L41e L44e
S6e S8e S17e S19e S24e S25e S26e S27Ae S28e S30e A LX
E
L6e L22e L28e L29e L36e L38e
S10e S21e
Large subunit (Haloarcula marismortui)
Small subunit (Thermus aquaticus)

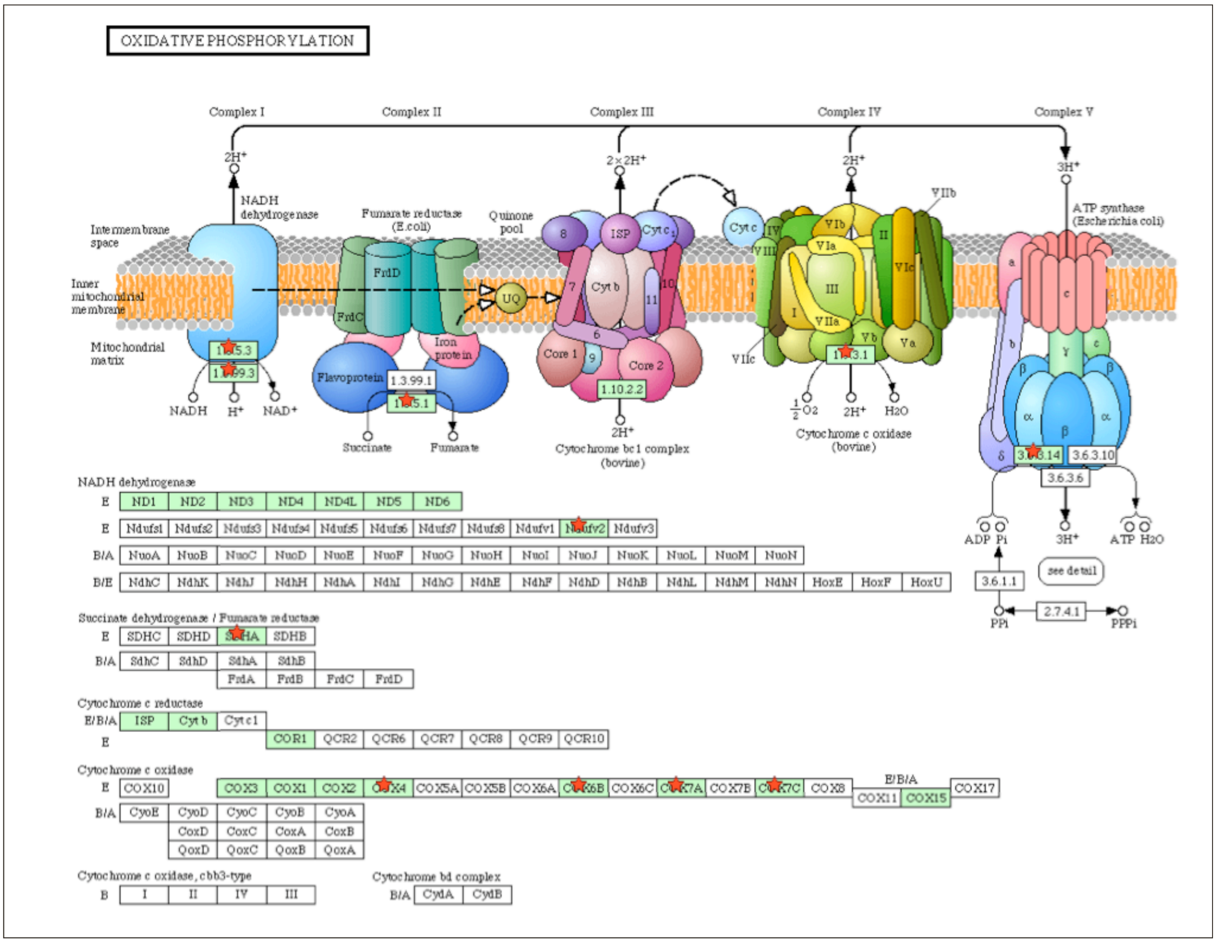
OXIDATIVE PHOSPHORYLATION
Complex I
Complex II
Complex III
Complex IV
Complex V
Intermembrane space
Inner mitochondrial membrane
Mitochondrial matrix
NADH dehydrogenase
Fumarate reductase (E.coli)
Quinone pool
Cytochrome bc1 complex (bovine)
Cytochrome c oxidase (bovine)
ATP synthase (Escherichia coli)
NADH H+ NAD+
Succinate Fumarate
Flavoprotein
Iron protein
Core 1 Core 2
Cyt b Cyt c1 ISP Cyt c
UQ
1.3.99.1
1.10.2.2
3.6.3.10
3.6.3.6
3.6.1.1
2.7.4.1
ADP Pi
ATP H2O
PPi PPPi
see detail
NADH dehydrogenase
E ND1 ND2 ND3 ND4 ND4L ND5 ND6
E Ndufs1 Ndufs2 Ndufs3 Ndufs4 Ndufs5 Ndufs6 Ndufs7 Ndufs8 Ndufv1 Ndufv2 Ndufv3
B/A NuoA NuoB NuoC NuoD NuoE NuoF NuoG NuoH NuoI NuoJ NuoK NuoL NuoM NuoN
B/E NdhC NdhK NdhJ NdhH NdhA NdhI NdhG NdhE NdhF NdhD NdhB NdhL NdhM NdhN HoxE HoxF HoxU
Succinate dehydrogenase / Fumarate reductase
E SDHC SDHD SDHA SDHB
B/A SdhC SdhD SdhA SdhB
FrdA FrdB FrdC FrdD
Cytochrome c reductase
E/B/A ISP Cyt b Cyt c1
E COR1 QCR2 QCR6 QCR7 QCR8 QCR9 QCR10
Cytochrome c oxidase
E COX10 COX3 COX1 COX2 COX4 COX5A COX5B COX6A COX6B COX6C COX7A COX7B COX7C COX8
E/B/A COX11 COX15 COX17
B/A CyoE CyoD CyoC CyoB CyoA
CoxD CoxC CoxA CoxB
QoxD QoxC QoxB QoxA
Cytochrome c oxidase, cbb3-type
B I II IV III
Cytochrome bd complex
B/A CydA CydB

PROTEASOME

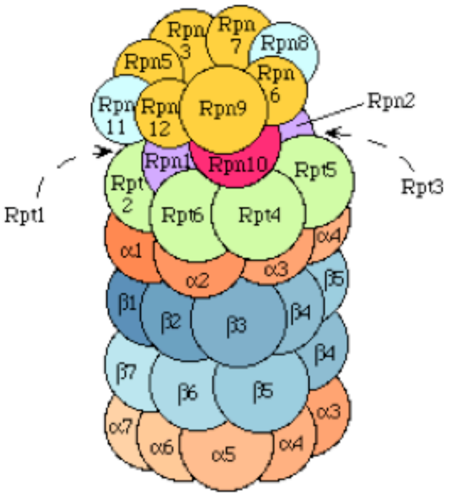

26S Proteasome (*Saccharomyces cerevisiae*)

Rpn1	Rpn2	Rpn3	Rpn4	Rpn5	Rpn6	
Rpn7	Rpn8	Rpn9	Rpn10	Rpn11	Rpn12	
Rpt1	Rpt2	Rpt3	Rpt4	Rpt5	Rpt6	
α1	α2	α3	α4	α5	α6	α7
β1	β2	β3	β4	β5	β6	β7
